# Inhibition of LIN28B impairs leukemia cell growth and metabolism in acute myeloid leukemia

**DOI:** 10.1186/s13045-017-0507-y

**Published:** 2017-07-11

**Authors:** Jianbiao Zhou, Chonglei Bi, Ying Qing Ching, Jing-Yuan Chooi, Xiao Lu, Jessie Yiying Quah, Sabrina Hui-Min Toh, Zit-Liang Chan, Tuan Zea Tan, Phyllis SY Chong, Wee-Joo Chng

**Affiliations:** 1 Cancer Science Institute of Singapore, National University of Singapore, Centre for Translational Medicine, 14 Medical Drive, Singapore, 117599 Republic of Singapore; 2 Department of Medicine, Yong Loo Lin School of Medicine, National University of Singapore, Singapore, 119074 Republic of Singapore; 3 Department of Hematology-Oncology, National University Cancer institute of Singapore, The National University Health System (NUHS), 1E, Kent Ridge Road, Singapore, 119228 Republic of Singapore

**Keywords:** LIN28B, let-7 microRNA, Acute myeloid leukemia (AML), Stem cell, Cancer metabolism, IGF2BP1

## Abstract

**Background:**

Current conventional chemotherapy for acute myeloid leukemia (AML) can achieve remission in over 70% of patients, but a majority of them will relapse within 5 years despite continued treatment. The relapse is postulated to be due to leukemia stem cells (LSCs), which are different from normal hematopoietic stem cells (HSCs). LIN28B is microRNA regulator and stem cell reprogramming factor. Overexpression of LIN28B has been associated with advance human malignancies and cancer stem cells (CSCs), including AML. However, the molecular mechanism by which LIN28B contributes to the development of AML remains largely elusive.

**Methods:**

We modulated LIN28B expression in AML and non-leukemic cells and investigated functional consequences in cell proliferation, cell cycle, and colony-forming assays. We performed a microarray-based analysis for LIN28B-silencing cells and interrogated gene expression data with different bioinformatic tools. AML mouse xenograft model was used to examine the in vivo function of LIN28B.

**Results:**

We demonstrated that targeting LIN28B in AML cells resulted in cell cycle arrest, inhibition of cell proliferation and colony formation, which was induced by de-repression of let-7a miRNA. On the other hand, overexpression of LIN28B promoted cell proliferation. Data point to a mechanism where that inhibition of LIN28B induces metabolic changes in AML cells. IGF2BP1 was confirmed to be a novel downstream target of LIN28B via let-7 miRNA in AML. Notably, ectopic expression of LIN28B increased tumorigenicity, while silencing LIN28B led to slow tumor growth in vivo.

**Conclusions:**

In sum, these results uncover a novel mechanism of an important regulatory signaling, LIN28B/let-7/IGF2BP1, in leukemogenesis and provide a rationale to target this pathway as effective therapeutic strategy.

**Electronic supplementary material:**

The online version of this article (doi:10.1186/s13045-017-0507-y) contains supplementary material, which is available to authorized users.

## Background

Acute myeloid leukemia (AML) is a heterogeneous clonal disorder characterized by the accumulation of differentiation-arrested myeloid progenitor cells (blasts) in the bone marrow and peripheral blood [1]. Sustained complete remission (CR) in AML has always been a challenge for oncologists despite much research on treatments has been done through deciphering the pathogenesis of this disease [2]. This underlying complexity is attributed to the fact that AML is not a single disease but a group of related diseases in which patients with different subtypes of AML vary in their outlook and response to treatment [3]. The development of targeted therapies holds promise for their potential towards a more effective and lower side effect treatment [4].

The relapse of AML is postulated to be due to existence of leukemia stem cells (LSCs) [5], which reside mostly in a quiescent cell cycle state that is similar to their normal hematopoietic stem cell counterparts, thus escaping from the effects of standard chemotherapy drugs which usually target proliferative cells [6]. LSCs are rare cells within tumors with the ability to self-renew and give rise to the phenotypically diverse tumor cell populations that result in tumorigenesis [7].

LIN28B is a stem cell reprogramming factor, downregulating let-7 microRNAs (miRNAs), where this occurs primarily at a post-transcriptional level [8]. Increased let-7 miRNAs promote stem cell differentiation [9]. Let-7 miRNA family consists of 13 members located in genomic locations, which are often mutated in human cancers [10–12]. However, the repression of let-7 miRNAs in cancer stem cells (CSCs) may be due to overexpression of LIN28B [13, 14].

LIN28B is highly expressed in embryonic stem cells, but in most differentiated adult tissues, it is significantly downregulated [15]. Overexpression of LIN28B has been associated with advance human malignancies [16], including leukemias [17, 18], and there are increasing evidences of its role in formation of CSCs and LSCs through either let-7 dependent or independent mechanisms [14, 15, 19]. Thus, given that LIN28B has a significant role in CSCs, it can serve as a vital target for eradication of CSCs, which are thought to be the origin of tumor resistance, recurrence, and metastasis of cancer [8].

We previously demonstrated that high expression of LIN28B is associated with worse survival in AML [18]. In this study, we determine the functional consequences and signaling network of LIN28B in AML through manipulating its expression. Our findings provide an advanced knowledge of LIN28B regulatory network in malignant hematopoiesis, as well as leukemia stem cell, thus providing a novel drug target for cancer therapy.

## Methods

### Cell culture

TF-1a, TF-1, HEL, and THP-1 AML cell lines were cultured in Roswell Park Memorial Institute (RPMI)1640 (Biowest, France), supplemented with 10% fetal bovine serum (Biowest) and 1% of 100× penicillin/streptomycin (P/S) (Biowest) at 37 °C with 5% CO_2_. Additional 4 ng/μl of recombinant human IL-3 (Preprotech, NJ, USA) was also added for TF-1. HEK-293T cells were maintained in Dublecco’s modified Eagle’s medium (DMEM) (Biowest) supplemented with 10% FBS and 1% of P/S at 37 °C with 5% CO_2_.

### Transfections and lentiviral shRNAs transductions

Polyethylenimine (PEI) (Sigma-Aldrich, MO, USA) was used for plasmid transfection of HEK-293T at 50–60% confluency. Protein or RNA was carried out 2 days after transfection. MyeloAim In Vitro Transduction reagent (BIOO Scientific, TX, USA) was used to transfect let-7 miRNA mimics to TF-1a cells. Cells were allowed to grow in MyeloAim medium complex containing 10 nM of let-7 miRNA mimic, 400 μl of serum free medium, and 40 μl of MyeloidAim reagent at 37 °C overnight before replacing the medium complex with complete growth medium and continued to culture for another 48 h. Plasmids pEGFP and LIN28B-pEGFP were transfected into TF-1 cells by Neon transfection (electroporation). TF-1 cells were resuspended with 100 μl of Resuspension buffer R (Invitrogen) per 1 × 10^6^ cells and incubated with 5 μg of plasmid. The Neon transfection system (Invitrogen) was performed at room temperature using 1200 voltage, 20 ms for three pulses. After transfection, cells were cultured in 1 ml of RPMI 1640 and incubated at 37 °C with 5% CO_2_.

#### Lentivirus production and transduction

The third generation packaging plasmids: pMDLg/pRRE, pRSV-Rev, and pMD2.G as described were transfected using an optimized ratio of 4 μg:2 μg:2 μg, respectively. Additional 5 μg of lentiviral LIN28B or LIN28B specific shRNAs constructs were added. LIN28B shRNA vectors were prepared according to the manufacturer’s instructions (Thermo Scientific). The five pairs of oligonucleotides used were human pLKO.1 lentiviral LIN28B-shRNA target gene set TRCN0000142983 (ShRNA1), TRCN0000143619 (ShRNA2), TRCN0000144508 (ShRNA3), TRCN0000219859 (ShRNA4), and TRCN0000219860 (ShRNA5). The five pairs of oligonucleotides used were human pLKO.1 lentiviral IGF2BP1-shRNA target gene set TRCN0000075148 (ShRNA1), TRCN0000075149 (ShRNA2), TRCN0000075150 (ShRNA3), TRCN0000075151 (ShRNA4), and TRCN0000075152 (ShRNA5). These plasmids were co-transfected using PEI to HEK-293T. Virus was harvested, pooled, and concentrated by centrifugation (Amicon). Infection of leukemia cells was done by the spin-infection method as described previously [20].

### Quantitative real-time RT-PCR

RNA was extracted using RNeasy Mini Kit (Qiagen Singapore) and performed according to the manufacturer’s protocol. One microgram of total RNA was used to perform cDNA synthesis using 4 μl of i-Script™ Reverse Transcription Supermix (Biorad, CA, USA). Two microliters of reaction products per reaction well (96-well plate) was used for real-time PCR with 0.8 μl of respective primers in 10 μl of PCR reagent (iTaq Universal SybrGreen mastermix, Biorad) to a total volume of 20 μl and performed under the following conditions: 2 min at 50 °C, 10 min at 95 °C, 15 s at 95 °C, and 1 min at 60 °C for 40 cycles using 7300 real-time PCR system (Applied Biosystems, CA, USA). GAPDH was used as the internal control. SDS 2.2.1 software (Applied Biosystems) was used to perform relative quantification of target genes using the comparative C_T_ (ΔΔC_T_) method. Quantification of let-7 miRNA was conducted with TaqMan MicroRNA assay using the same 7300 real-time PCR system protocol as described earlier [18]. The primer sequences were described in Additional file 1: Table S1.

### Cell viability assays

Leukemic cells were seeded in 96-well culture plates at a density of 2 × 10^4^ viable cells/100 μl/well in triplicates. CellTiter-Glo® Luminescent Cell Viability Assay (CTG assay, Promega, Madison, WI) was used to determine the cell growth and viability as previously described. Each experiment was in triplicate.

### Cell cycle analysis

Total 5 × 10^5^ cells/well of cells were labeled in medium containing 10 μM of BrdU (Becton Dickinson, NJ, USA) in 12-well plates and then cultured for 12 h. Cells were stained with Aqua dye (Gift from Dr. Osato Motomi’s lab) for 30 min prior fixation. Then, APC-anti-BrdU (Becton Dickinson) and 7-Aminoactinomycin D (7-AAD) (Becton Dickinson) were used according to the manufacturer’s protocol. Samples were analyzed using BD LSR II flow cytometer (Becton Dickinson) and Flowjo (Treestar, OR, USA). Cell cycle profiles were determined based on the 7-AAD/anti-BrdU staining pattern, while non-viable cells were identified and excluded based on the excitation level from aqua dye. We also analyzed DNA histograms for cell cycle using propidium iodide (PI)/RNase staining buffer (BD Pharmingen, San Diego, CA).

### Protein extraction and Western blotting

Total protein extracts were vortex and prepared in lysis buffer (1% Nonidet P-40, 50 mM Tris, pH 8.0, 50 mM NaCl, 1 mM EDTA, 10% glycerol, protease and phosphatase inhibitors). The samples were separated on 12% polyacrylamide denaturing gels and transferred to PVDF membranes. Membranes were saturated for 1 h in 5% milk with 0.1% Tween 20-PBS (PBS-T) solution before incubating with the following antibodies: LIN28B antibody, IGF2BP-1 antibody, Cyclin B1 antibody, p21waf1 antibody from Cell Signaling Technologies (Danvers, MA) and Cyclin D1 antibody, beta-actin HRP conjugated antibody from Santa Cruz Biotechnology (Santa Cruz, CA).

### Affymetrix microarray analysis

TF-1a cells were transduced with either Scramble shRNA or LIN28B-shRNA5 for 72 h. Cells were harvested, and total RNA was extracted using the RNeasy Mini Kit, according to the manufacturer’s instruction (Qiagen). RNA quantity, quality, and purity were assessed with the use of the RNA 6000 Nano assay on the Agilent 2100 Bioanalyzer (Agilent Technologies, Santa Clara CA, USA).

Gene expression profiling was performed using Affymetrix U133plus2.0 gene chip (Affymetrix, Santa Clara, CA) according to the manufacturer’s protocol. The scanned data was processed using MicroArray Suite version 5.0 (MAS) (Affymetrix). The gene expression data was then log-transformed, median centered, and analyzed using the Genespring GX 7.3.1 software (Agilent Technologies, Santa Clara CA). Sequential filtering was employed to select genes for subsequent analysis. First, non-expressed probe sets (assigned an absent or marginal flag by MAS) were excluded. The remaining probe sets were subjected to ANOVA across the samples. The probe sets with significant variation with a corrected *p* value of less than 0.05 after multiple testing corrections using the Benjamini and Hochberg methods were used for subsequent comparative analysis.

### Gene ontology (GO) and IPA pathway analysis of differentially expressed genes

GO analysis of the significant probe list was performed using PANTHER (http://www.pantherdb.org/), using text files containing the Gene ID list and accession numbers of the Affymetrix probe ID. The same list of differentially expressed genes was input into Ingenuity Pathway Analysis (IPA) (Ingenuity Systems; Mountain View, CA, USA). A comprehensive search to identify their biological functions, gene interaction networks, and pathway analysis was conducted by IPA system. The identified genes were mapped to genetic networks available from the Ingenuity database and were then ranked by score. The significance was set at a *p* value of 0.05.

### Measurements of metabolites

TF-1a-Scramble, TF-1a-LIN28B-shRNA3, and TF-1a-LIN28B-shRNA5 cells were lysed in RIPA lysis buffer. Three kits were purchased from Abcam (Cambridge, UK), including Glutamate Assay Kit (Fluorometric) (ab138883), L-Amino Acid Assay Kit (ab65347), and Aspartate Assay Kit (ab102512). The measurement of glutamate, L-amino acid, and aspartate were performed according to manufacturer’s specifications.

### AML xenograft model

Six-week-old female nonobese diabetic/severe combined immunodeficient (NOD/SCID) mice were purchased from In Vivos Singapore. Exponentially growing TF1-pEGFP, TF1-LIN28B cells (3 × 10^6^), as well as TF1-LIN28B cells expressing LIN28B-shRNA5 cells (TF1-LIN28B-sh5) were mixed with Matrigel (50%) and subcutaneously injected into loose skin between the shoulder blades and the left hind leg of NOD/SCID-recipient mice, respectively. Each group has 10 mice. The length (L) and width (W) of the tumor were measured with calipers every 2 days, and tumor volume (TV) was calculated as TV = (L × W^2^)/2. At the end of experiments, mice were euthanized and tumors were dissected. The protocol is reviewed and approved by Institutional Animal Care and Use Committee in compliance to the guidelines on the care and use of animals for scientific purpose.

## Results

### LIN28B regulates cancer cells proliferation

**Fig. 1:**
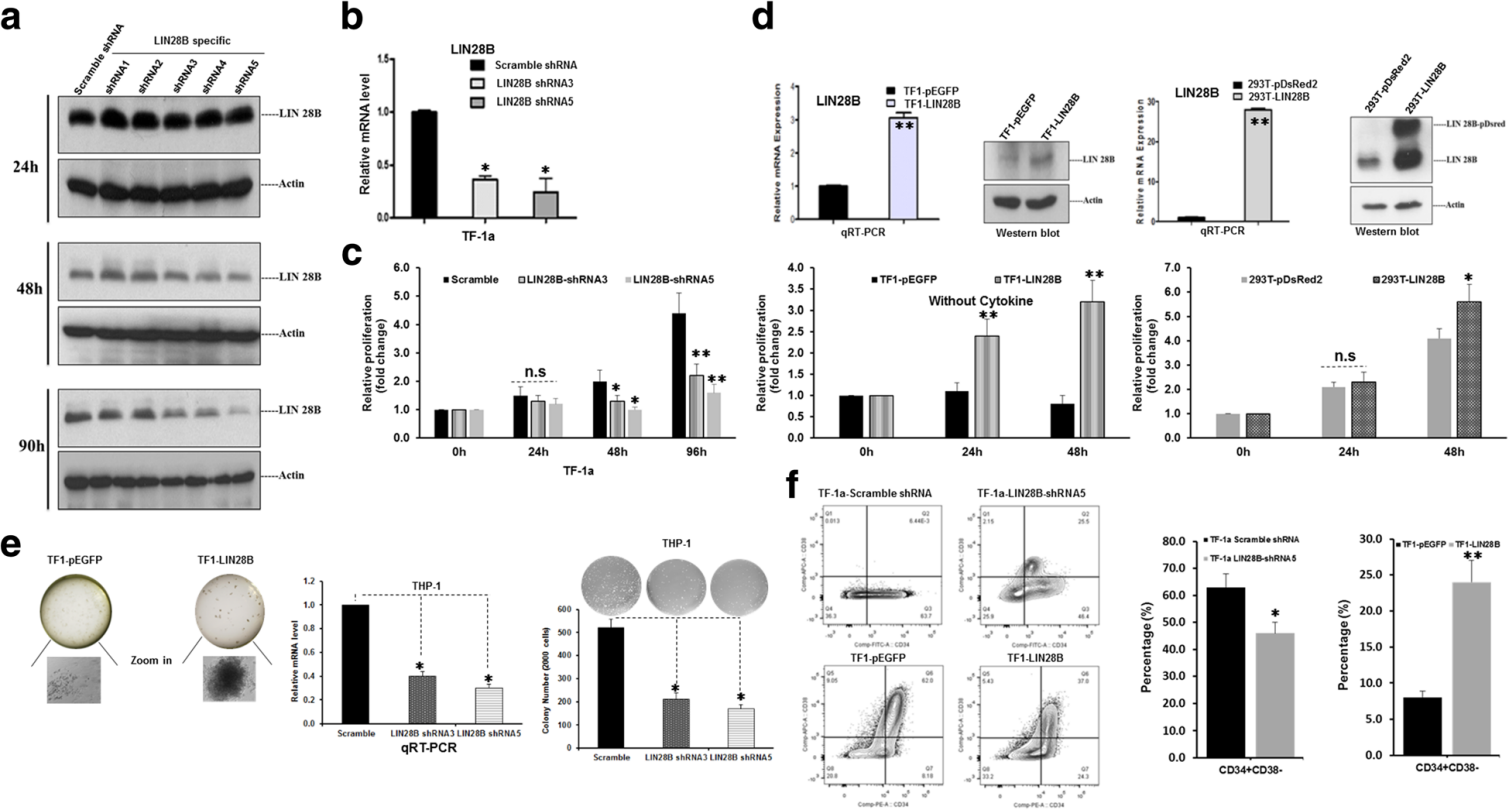
The effect of silencing LIN28B in AML. **a** Lentiviral LIN28B shRNA 1, 2, 3, 4, 5 and Scramble-shRNA were transduced in TF-1a cells. Proteins of the knockdown cell lines were harvested at 24, 48, and 90 h time points for Western blot analysis. β-actin was used as a loading control. **b** RNAs of shRNA 3 and 5 transduced cells were extracted after 1 week of drug selection. qRT-PCR was performed to compare LIN28B transcription level. The expression of LIN28B in each sample was normalized with GAPDH, respectively (*n* = 3, mean ± SD). *p < 0.01. **c** Cell viability assay of TF-1a cells transduced with Scramble shRNA or LIN28B shRNA 3, 5. Eight repeats of 10,000 cells in 100 μl of medium each per sample were cultured at various time points: 0, 24, 48, and 96 h by CTG assays. Measurements taken from 24 h onwards were normalized with their respective values obtained at 0 h (*n* = 8, mean ± SD). *n.s.* not significant; *p < 0.05; **p < 0.01. **d** qRT-PCR and Western blot analysis validation of overexpression of LIN28B in TF-1 and HEK293T cells, followed by cell viability assays at 0, 24, and 48 h times. Measurements taken from 24 h onwards were normalized with their respective values obtained at 0 h (*n* = 8, mean ± SD). *n.s.* not significant; *p < 0.05; **p < 0.01. **e** Colony-forming assay of TF1-pEGFP and TF1-LIN28B. The experiments were duplicated and representative pictures were presented (*left panel*). qRT-PCR analysis confirmed decreased LIN28B expression in LIN28B shRNA3, 5 treated THP-1 cells (*middle panel*). Quantification of colonies of indicated cell lines (*n* = 3, mean ± SD). The colony number of LIN28B-shRNAs expression THP-1 cells was significantly reduced than those of Scramble shRNA expressing cells (*right panel*, *p < 0.01). **f** Flow cytometric detection of the CD34^+^CD38^−^ population in pairs of LIN28B knockdown and overexpression cell lines. The representative FACS images were shown in the *left panel* and *bar graphs* of the percentages of CD34^+^CD38^−^ cells were listed in the *right panel* (*n* = 2, mean ± SD) (*p < 0.05, **p < 0.01)

TF-1a AML cell line, a more immature and aggressive phenotype of leukemia, shows increased LIN28B expression [18, 21]. In order to study the functional effect of LIN28B, five shRNAs specific targeting LIN28B were transfected into the TF-1a cells and to determine their knockdown efficiencies. After transfection 24 h, there was no difference in the level of LIN28B expression. However, LIN28B protein was remarkably decreased post transfection 48 and 96 h in LIN28B-shRNA3, 4, and 5 transfected cells, while shRNA1 and 2 could not achieve the desired knockdown result (Fig. 1a). The reduction of LIN28B mRNA by LIN28B-shRNA3 and 5 was evaluated by qRT-PCR too. The results showed that both shRNA 3 and 5 could reduce LIN28B mRNA levels by 76 and 65.5%, respectively (Fig. 1b).

We then quantify the cells viability of TF-1a and TF-1a-LIN28B knockdown cells. Both LIN28B-shRNA3 and 5 transduced TF-1a cells showed decreased cell proliferation than TF-1a cells, indicating that LIN28B regulates cell proliferation. The difference in their proliferation varied by around 1.5- to 2.8-fold in 24 through 96 h (Fig. 1c). To exclude nonspecific effect, we overexpressed LIN28B in TF-1 and HEK293T cells, a non-leukemia cell line. LIN28B overexpression was confirmed on both mRNA and protein level in these two cell lines (Fig. 1d). LIN28B mRNA was increased threefold in TF-1 cells overexpressing LIN28B-pEGFP plasmid relative to vector control cells. This pair of cells were then cultured without the addition of cytokine and CTG assay were done at different time points. The result showed that TF1-pEGFP cells did not proliferate in absence of cytokine; however, TF1-LIN28B cells continue to grow well at 24 and 48 h (Fig. 1d). These data suggested that cytokine dependency could be abrogated by increasing LIN28B protein expression.

To exclude cell type-specific effect, HEK293T cells were transfected with LIN28B-pDsRed2 plasmid and empty vector pDsRed2. qRT-PCR result showed that LIN28B mRNA was overexpressed by 30-fold as compared to the control (Fig. 1d). Validation by Western blot also depicts a corresponding increase in LIN28B-pDsRed and endogenous LIN28B proteins (Fig. 1d). The high expression of LIN28B-pDsRed might result in further repression of let-7, thus inhibiting let-7 function might explain the increase of endogenous LIN28B protein. Indeed, overexpression of LIN28B could also lead to enhanced cell proliferation in *293T* cells (Fig. 1d).

**Fig. 2:**
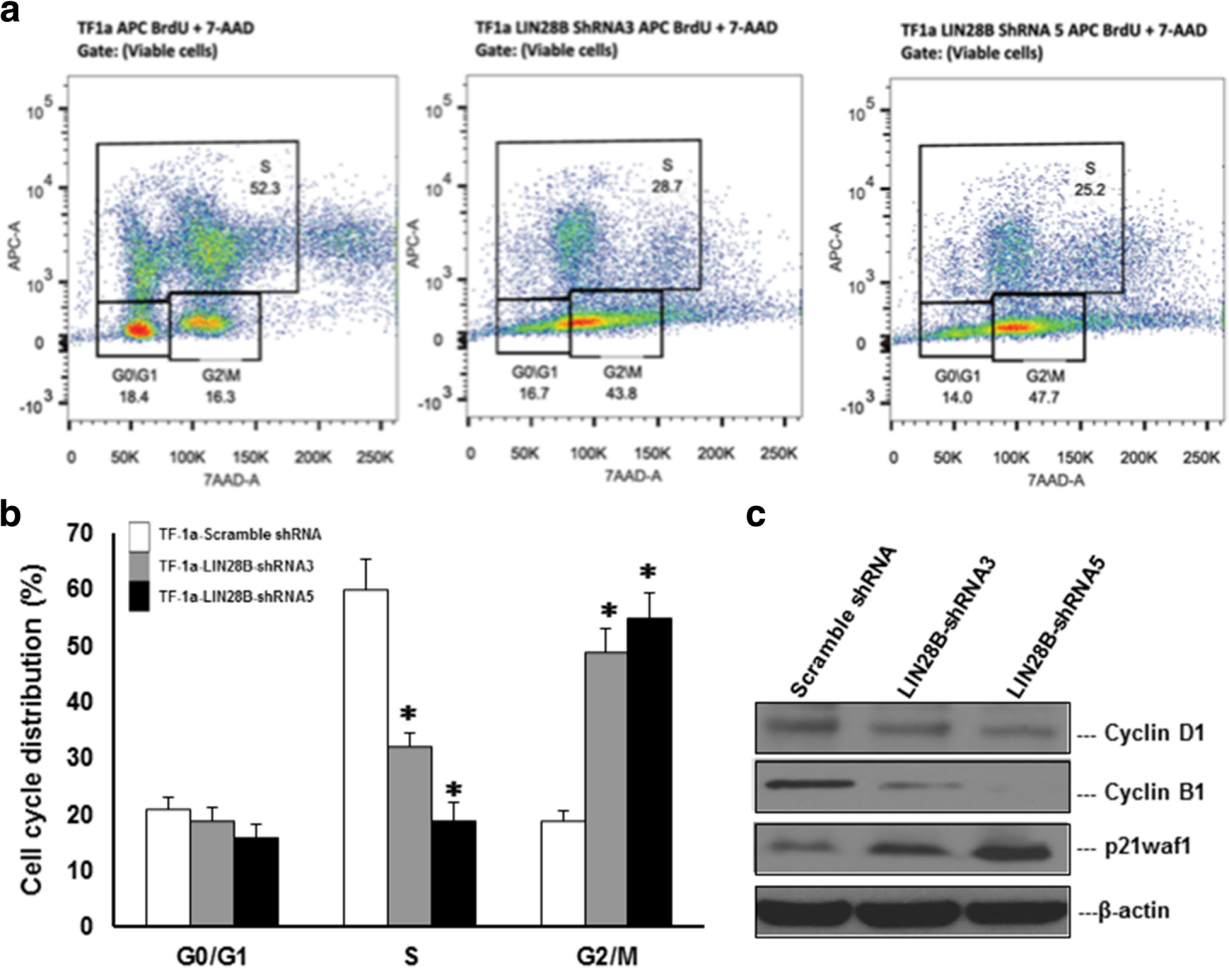
LIN28B regulates cell cycle progress of AML cells. TF-1a cells were treated either with Scramble shRNA or LIN28B shRNA3 and 5, then grown with BrdU for 12 h stained with aqua dye prior fixation and addition of anti-BrdU APC and 7-AAD as described in Materials and methods section. **a** Representative images of how viable cells were identified, gated and plot as pseudocolour dot-plots with APC versus 7-AAD. **b** Representative data showed the distribution of cells at each stage of the cell cycle (in percentage) determined by flow cytometric analysis. This experiment was duplicated. (*n* = 2, mean ± SD, *p < 0.05; **p < 0.01). **c** The cell lysates extracted from TF-1a-Scramble shRNA, TF-1a-LIN28B-shRNA3, and TF-1a-LIN28B-shRNA5 were subjected to Western blot analysis for Cyclin D1, Cyclin B1, p21waf1, and beta-actin. Beta-actin was used as internal control

Furthermore, in vitro clonogenic capacity is one of the hallmarks of LSCs and it is well established that LIN28B plays an important role in CSCs and cellular reprogramming. Therefore, methylcellulose assay showed only TF1-LIN28B cells, but not TF1-pEGFP cells, formed colonies (Fig. 1e). We also applied LIN28B-shRNA3 and 5 to selectively knockdown LIN28B expression in THP-1 AML cell line, followed by CFU assays. As shown in Fig. 2e, qRT-PCR analysis confirmed that significant reduction of LIN28B level in THP-1 cells. Indeed, we observed the colony number of LIN28B-shRNAs expressing THP-1 cells was markedly decreased relative to scramble shRNA-treated cells respectively (Fig. 1e). It is well established that LSCs are particularly enriched in the CD34^+^CD38^−^ cell population of AML cells [6, 22–24]; we set to investigate the fluctuations of this particular population after modulating LIN28B expression. The FACS data revealed that the CD34^+^CD38^−^ were significantly decreased in the TF-1a-LIN28-shRNA5 expressing cells, constituting an average of 46% (*p* < 0.05 versus the Scramble-shRNA cells: average 64%) of the total cells (Fig. 1f). Consistently, the TF1-LIN28B cells contained about 3 times more CD34^+^CD38^−^ subpopulation than TF1-pEGFP cells had (*p* < 0.01) (Fig. 1f). While it remains to be proven in vivo, these results collectively strength the conclusion that LIN28B is one of the key regulators of LSC properties of AML cells.

Taken together, these evidence demonstrate an important role of LIN28B protein in AML cell proliferation and colony formation, thus potentially conferring cytokine-independent growth leukemic cells.

### Inhibition of LIN28B induces G_2_/M cell-cycle arrest

We investigated whether cell cycle was affected in LIN28B knocking down cells. Figure 2a depicted pseudocolour dot plots of the analyzed cells, which both LIN28B-shRNA expressing TF-1a cells have lower BrdU incorporation than the wild type, suggesting that the cells were less active to undergo cell division. Meanwhile, increased percentage of cells was found on G_2_/M quadrant, implying that G_2_/M cell cycle arrest of LIN28B-shRNA expressing cells. A bar chart (Fig. 2b) shows BrdU incorporation (S phase) of LIN28B knocking down TF-1a cells were significantly lower (32% for TF-1a LIN28B shRNA3 and 29% for TF-1a LIN28B ShRNA 5) than TF-1a, which is 60% (*p* < 0.01). G_2_/M populations were 49 and 55% for both LIN28B knockdown cells, while only 19% in the normal TF-1a cells (*p* < 0.01). FACS analysis of DNA histograms confirmed the observations of G_2_/M cell cycle arrest and S phase inhibition after LIN28B knockdown (Additional file 2: Figure S1). The cell cycle is a precisely regulated by different sets of cyclins and cyclin-dependent kinases (CDKs). Therefore, we further evaluated the levels of the cyclin proteins to examine whether their expression levels were associated with LIN28B knockdown-mediated G_2_/M arrest. As shown in the Fig. 2c, cyclin D1 and cyclin B1 were notably reduced, associated with upregulation of the CDK inhibitor p21waf1 in the two TF-1a cells expressing LIN28B shRNAs. Overall, these results correlate with data in Fig. 1a where the greater the reduction of LIN28B in shRNA5, the higher percentage of the population in G_2_/M phase was arrested.

### LIN28B controls the expression of genes involved in metabolic processes

**Fig. 3:**
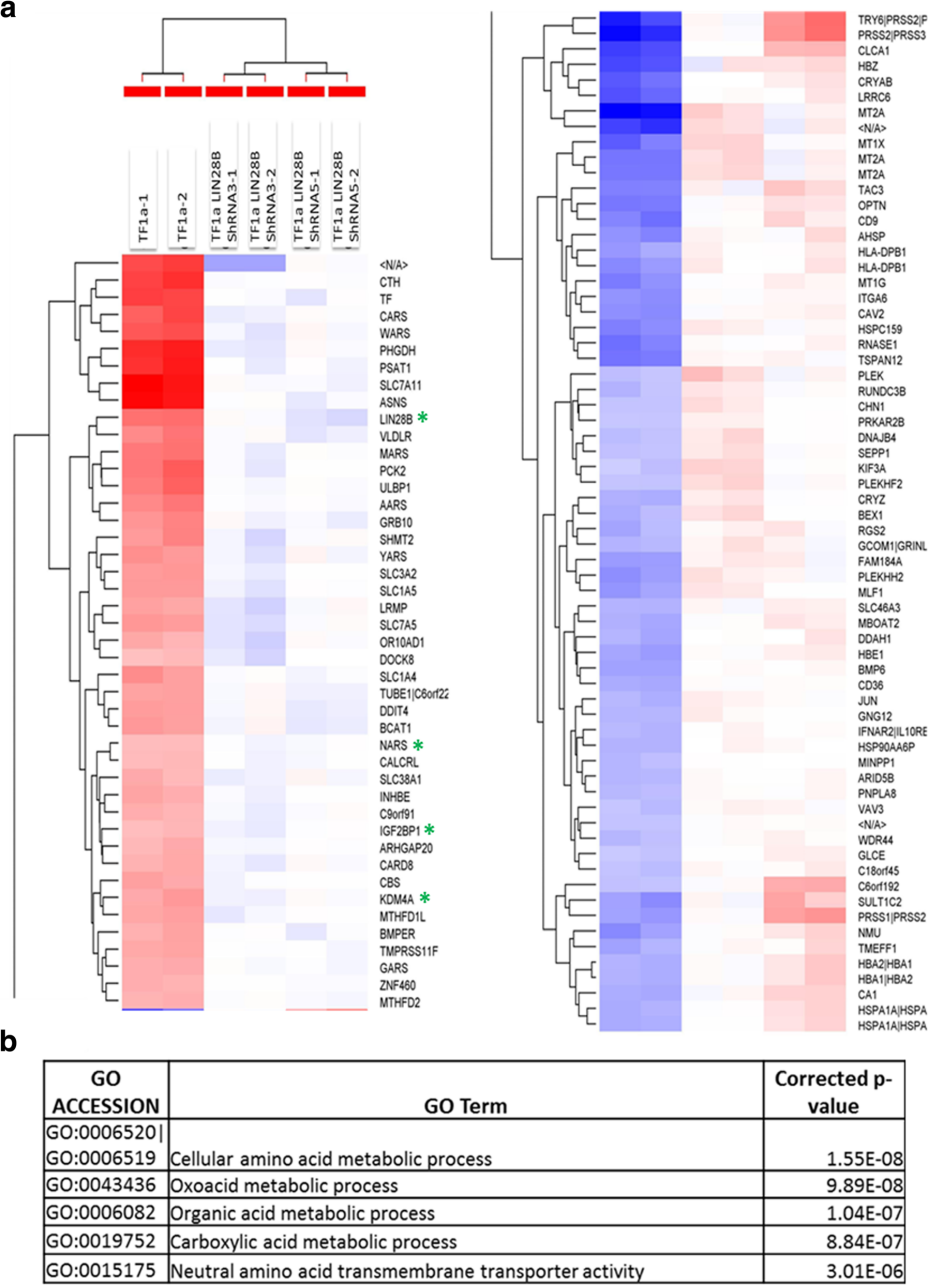
Microarray analysis of LIN28B knocking down TF-1a cells. **a** Relative expression levels for the 108 genes that changed significantly (ANOVA, *p* < 0.05) and more than 1.4-fold are shown in six columns. *Colors* indicate relative signal intensities (red: gene upregulation; blue: gene downregulation). The RNA expression profile was sorted using a hierarchical clustering method. A quantitative description of RNA expression levels is presented in Additional file 1: Table S1. Duplication was done and shown in this figure. N/A indicates unknown gene which its sequence might have potential biological effect. **b** GO analysis revealed that these top five most significant gene cluster variation in LIN28B knocking down TF-1a cells

**Fig. 4:**
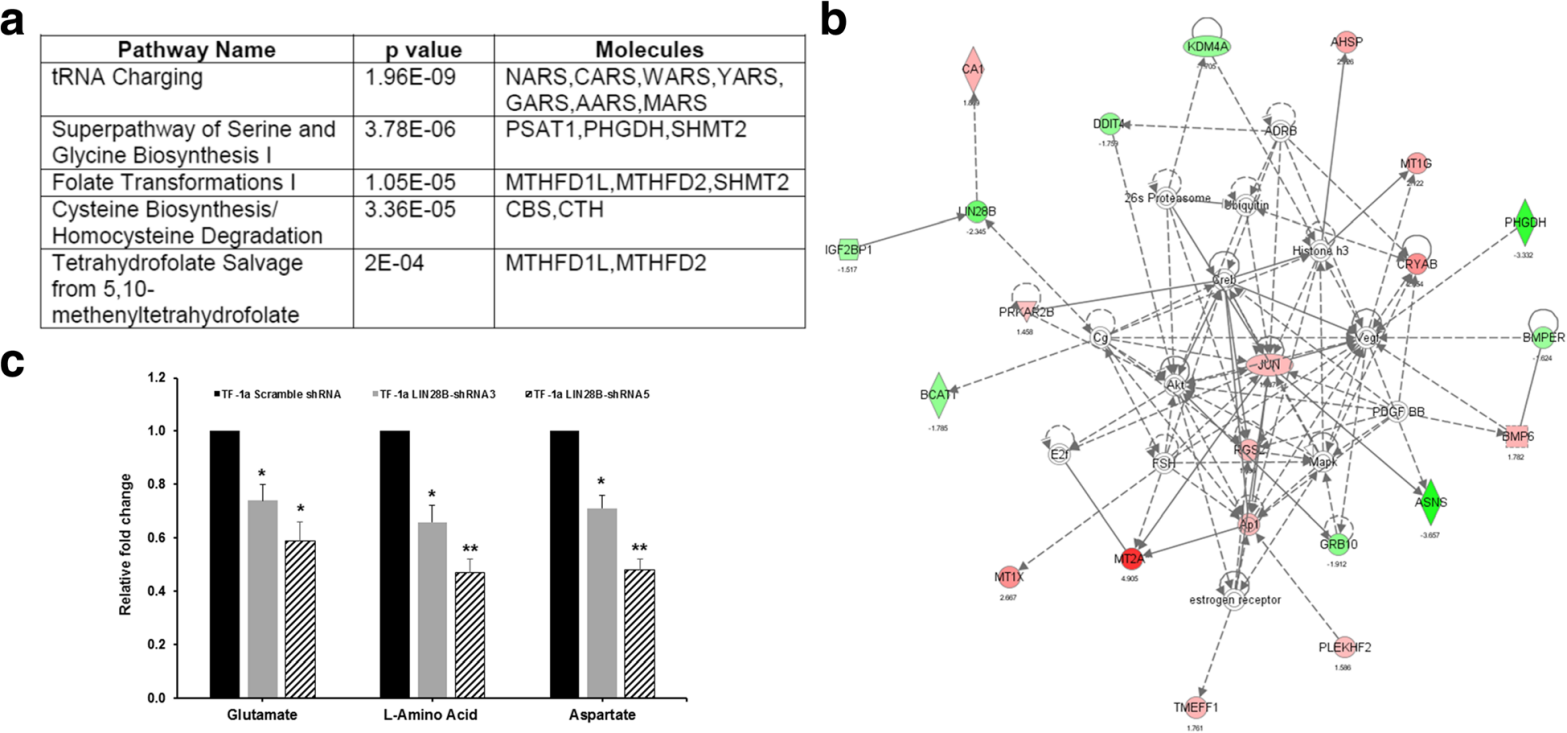
Networks predicted by Ingenuity Pathway Analysis in the LIN28B knocking down cells. **a** Top five canonical pathways affected by silencing LIN28B. **b** The interaction network displayed graphically as nodes (genes) was centered on JUN and LIN28B. The *coding color* and *lines* are defined by IPA software by default. The *node color intensity* indicates the expression of genes, with *red* representing upregulation and *green* representing downregulation. *Solid lines* and *dotted lines* indicate direct relationship and indirect relationships, respectively. **c** The measurements of glutamine, l-amino acid, and aspartate in TF-1a-Scramble shRNA, TF-1a-LIN28B-shRNA3, and TF-1a-LIN28B-shRNA5 cell lysates. The relative fold changes were compared to the amount of these three metabolites in TF-1a-Scramble shRNA cell lysate, which were set as 1. The experiments were triplicated (mean ± SD). *p < 0.05; **p < 0.01

To gain insight into the role of LIN28B in leukemogenesis, we conducted an mRNA expression microarray analysis of TF-1a cells expressing Scramble shRNA or LIN28B-shRNA3 and 5. Using 1.4-fold as cut-off level, 64 genes showed increased expression and 44 genes had decreased expression in both TF-1a LIN28B-shRNA3 and 5 expressing cells compared to TF-1a-Scramble shRNA cells (Fig. 3a). These differential expressions were statistically significant (*p* < 0.05, the complete list shown in Additional file 3: Table S2). The reduction of LIN28B expression in knocking down cell lines matched with the previous qRT-PCR result (Fig. 1b), thus indicating the knockdown was successful and also validates the credibility of this microarray data. Notably, some important oncogenes, such as KDM4A, PSAT1, and IGF2BP1, were decreased in LIN28B shRNAs-treated cells (Fig. 3a and Additional file 3: Table S2). GO term analysis revealed that genes involved in metabolic processes and neutral amino acid transmembrane transporter activity showed the most significant change (Fig. 3b). We then performed IPA, which identified top five canonical pathways from these differentially expressed genes, based on the literature contained in the IPA knowledge base (*p* < 0.05) (Fig. 4a). Except tRNA charging, the other four pathways were all related with metabolism, including Superpathway of Serine and Glycine Biosynthesis I, Folate Transformations I, Cysteine Biosynthesis/Homocysteine Degradation, and Tetrahydrofolate Salvage from 5,10-methenyltetrahydrofolate. This analysis also revealed one interaction map associated with JUN (upregulated gene) and LIN28B itself, which were central genes (Fig. 4b). In addition to LIN28B and IGF2BP1, qRT-PCR analysis also confirmed the decreased expression level for PSAT1 PHGDH SHMT2 MTHFD1L MTHFD2 CBS CTH KDM4A and increased level for JUN genes (Additional file 4: Figure S2), which were consistent with the microarray results. Moreover, to quantify the differences in the amount of some metabolites caused by LIN28B, the amount of glutamate, l-amino acid, and aspartate was measured by fluorometric assay. The quantities of these three metabolites were significantly lower in TF-1a-LIN28 shRNA expressing cells than in the Scramble shRNA expression cells (Fig. 4c, *p* < 0.05). Notably, the degree of reduction of metabolites was correlated with the LIN28B protein knocking down level (Fig. 1a).

Taking consideration of GO term analysis together, these data pinpoint the role of LIN28B in promoting cell proliferation and survival might occur through downstream regulation of metabolism and signaling pathways.

### IGF2BP1 is a downstream effector of LIN28B through let-7 miRNA

**Fig. 5:**
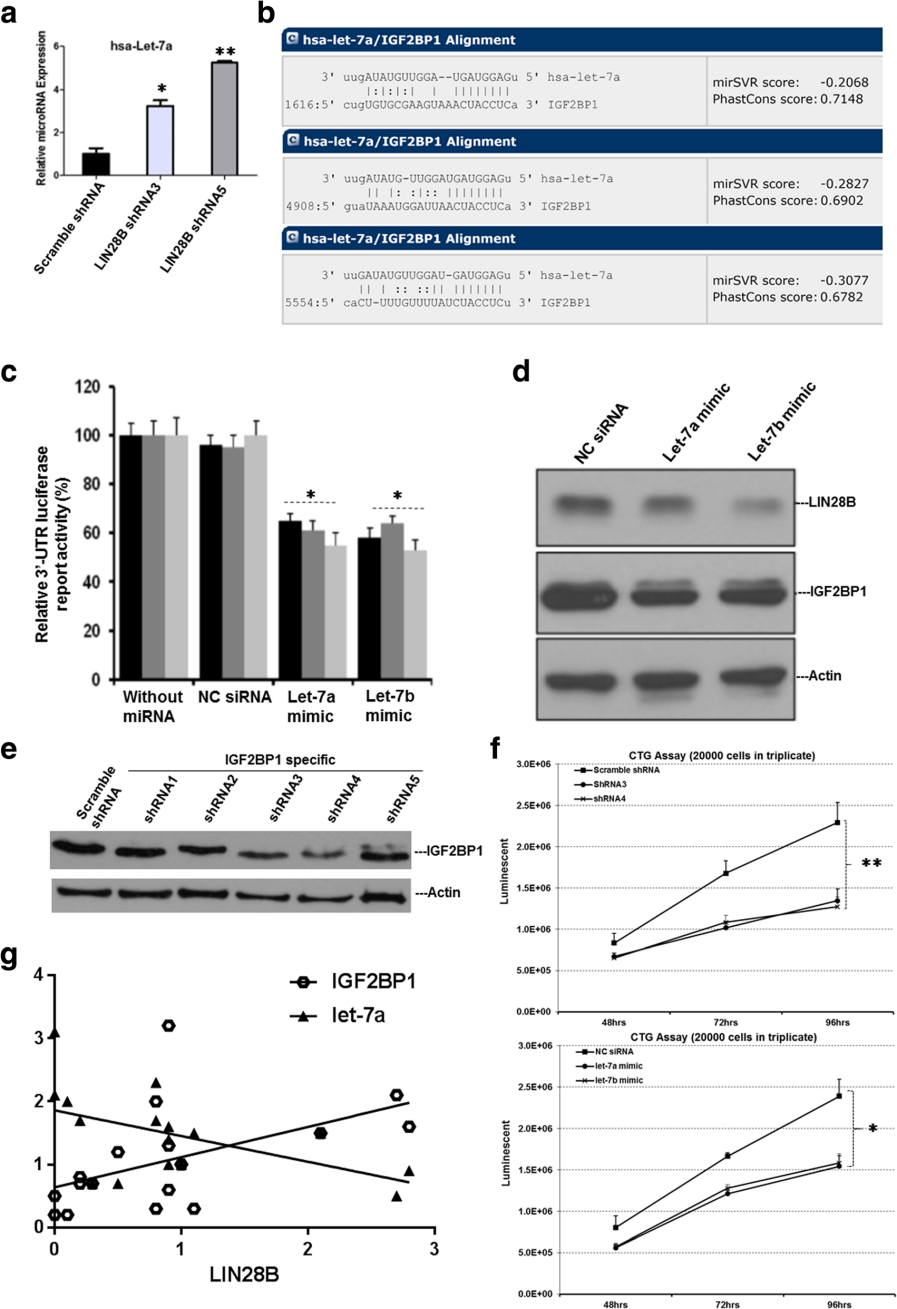
Characterization of IGF2BP1 as downstream target of LIN28B via let-7 microRNA. **a** Relative let-7a microRNA expression of TF-1a after LIN28B knockdown as quantified by qPCR. *p < 0.05; **p < 0.01. **b** Potential let-7 miRNA binding sequence of 3′-UTR of IGF2BP1. **c** HEK293T cells were co-transfected with 0.5 μg of pGL3.0, 2 μg of IGF2BP1 reporter gene, and 10 ng of Renilla vector as indicated on the *x*-axis in 1 ml of RPMI 1640 growth medium for 24 h prior to lysis for measuring the luminescence. All samples were normalized with renilla luciferase to ensure equal transfection efficiency. Cells transfected with control siRNA (NC siRNA) and without miRNA were used as negative controls. **d** let-7a and let-7b mimetic were chemically transfected into TF-1a cells and cultured for 72 h prior protein extraction for Western blot analysis. **e** Assessment of IGF2BP1 protein levels in HEL cells transduced with Scramble shRNA or IGF2BP1 specific shRNAs after 72 h. **f** Cell proliferation assays of HEL cells treated with IGF2BP1-specific shRNA3, shRNA4 (*left panel*) or let-7a mimic, let-7b mimics at indicated time points (*n* = 3, mean ± SD, *p < 0.05, **p < 0.01). **g** LIN28B expression primary AML cell was correlated with increased IGF2BP1 and decreased let-7a. Bone marrow samples from 17 patients with AML were collected at diagnosis and subjected to qPCR for LIN28B, IGF2BP1, and let-7a miRNA levels. The Pearson *r* values and *p* values were determined with GraphPad Prism software

The reciprocal regulations between LIN28B and let-7 have been confirmed in many studies [16, 25, 26]. Cells with high level of LIN28B are regarded to have low expression of let-7 miRNA and vice versa. Consistently, qRT-PCR result showed a significant increase in let-7a miRNA expression in both LIN28B shRNA expressing TF-1a cells. These changes were inversely proportional to LIN28B knockdown (Fig. 5a), in that, TF-1a cells expressing LIN28B-ShRNA5, which displayed a stronger LIN28B reduction than those expressing LIN28B-ShRNA3, had a higher let-7a miRNA expression. In conclusion, these data indicate LIN28B negatively regulates let-7a miRNA.

We and others reported previously that overexpression of LIN28B contributes to tumor aggressiveness and metastasis through let-7-dependent mechanisms [16, 18]. Recently, we discovered that both of insulin-like growth factor 2 mRNA-binding protein 1 (IGF2BP1) and LIN28B were upregulated by PRL-3 through microarray study. IGF2BP1 acts as oncogene in many types of solid tumors, and it has been identified as a novel IgH translocation partner in B acute lymphoblastic leukemia (ALL). Furthermore, IGF2BP1 was decreased after silencing LIN28B in TF-1a cells as revealed by gene expression profiling (Fig. 3a and Additional file 3: Table S2). Putting together, these evidence imply a close relationship between LIN28B and IGF2BP1. But how they exactly regulate each other in AML is not well understood. Bioinformatics predictions from DIANAmT, miRDB, miRwalk, PICTAR5, and Targetscan databases revealed IGF2BP1 as a potential target for the let-7 family miRNAs (Table 1). Subsequently, target sequence predictions using database from microrna.org suggested three possible targets where let-7a could bind onto (Fig. 5b). Next, the three 3′-UTR of IGF2BP1 sequences were cloned into pGLO reporter vectors and expressed individually in HEK 293 cells. As shown in Fig. 5c, transfection of the cells with let-7a and let-7b mimics led to a significant reduction in the reporter activity of IGFBP1 3′-UTR sequence as compared to the negative controls, suggesting that IGF2BP1 could be downregulated in vitro by overexpression of let-7a and let-7b miRNAs. Western blot analysis of TF-1a cells that transfected with let-7a and let-7b mimics further confirmed that IGF2BP1 could be downregulated by let-7a and let-7b, in addition to simultaneously reduced LIN28B expression as there is a feedback regulatory loop between LIN28B and let-7 miRNAs (Fig. 5d) [8]. To examine the functional importance of IGF2BP1 in LIN28B-mediated leukemogenesis, we used HEL AML cell line with endogenous LIN28B to study loss-of-function phenotype by way of depletion of IGF2BP1 or addition of let-7 mimics. Total five lentiviral shRNAs particles specific targeting IGF2BP1 were transduced into the HEL cells and their knockdown efficiencies after 72 h were determined. The success of knockdown of IGF2BP1 by two independent shRNA3 and shRNA4 was confirmed by Western blot analysis (Fig. 5e). Then, these two IGF2BP1-knockdown cell lines were subjected to CTG assays. In fact, loss-of-IGPF2BP1 or additional let-7 mimics led to about a 1.8-fold or 1.5-fold reduction in proliferation, respectively (Fig. 5f, *p* < 0.01 or *p* < 0.05, respectively). We next applied similar approaches to examine the effects of silencing IGF2BP1 or additional let-7 miRNA mimics in a different cell line, TF-1a cell line. In agreement with the results from HEL cells, silencing of IGF2BP1 or supplying let-7 miRNA mimics significantly suppressed the growth rates of TF-1a cells (Additional file 5: Figure S3). In order to further validate this pathway in patients, we analyzed and compared the expression levels of Lin28B, IGF2BP1, and let-7a in 17 bone marrow samples from de novo AML patients. qRT-PCR analysis (Fig. 5g) showed that a significant positive correlation between the expression of LIN28B and IGF2BP1 (Pearson *r* = 0.513; *p* = 0.035) and a negative correlation between LIN28B and let-7a (Pearson *r* = 0.534; *p* = 0.027).

**Table 1:** Bioinformatic predictions of a common target by let-7 family

Gene	miRNA	StemLoopID	DIANAmT	miRanda	miRDB	miRWalk	PICTAR5	Targetscan	Sum
IGF2BP1	hsa-let-7a	hsa-let-7a-3	1	1	1	1	1	1	6
IGF2BP1	hsa-let-7a	hsa-let-7a-1	1	1	0	1	0	1	4
IGF2BP1	hsa-let-7a	hsa-let-7a-2	1	1	0	1	0	1	4
IGF2BP1	hsa-let-7b	hsa-let-7b	1	1	1	1	1	1	6
IGF2BP1	hsa-let-7c	hsa-let-7c	1	1	1	1	1	1	6
IGF2BP1	hsa-let-7c^*^	hsa-let-7c	1	1	0	0	0	0	2
IGF2BP1	hsa-let-7d	hsa-let-7d	1	1	1	1	1	1	6
IGF2BP1	hsa-let-7e	hsa-let-7e	1	1	1	1	1	1	6
IGF2BP1	hsa-let-7e^*^	hsa-let-7e	1	0	0	0	0	0	1
IGF2BP1	hsa-let-7f	hsa-let-7f-2	1	1	1	1	1	1	6
IGF2BP1	hsa-let-7g	hsa-let-7g	1	1	1	1	1	1	6
IGF2BP1	hsa-let-7i	hsa-let-7i	1	1	1	1	1	1	6
IGF2BP1	hsa-let-7i*	hsa-let-7i	1	0	0	0	0	0	1
IGF2BP1	hsa-miR-98	hsa-mir-98	1	1	1	1	1	1	6

*the minor-strand (star-strand)

Hence, these results indicate that IGF2BP1 is modulated in a let-7-dependent mechanism and that overexpression of LIN28B would suppress let-7 miRNAs, thereby increasing IGF2BP1 protein expression in AML cells.

### LIN28B enhances tumorigenicity in vivo

**Fig. 6:**
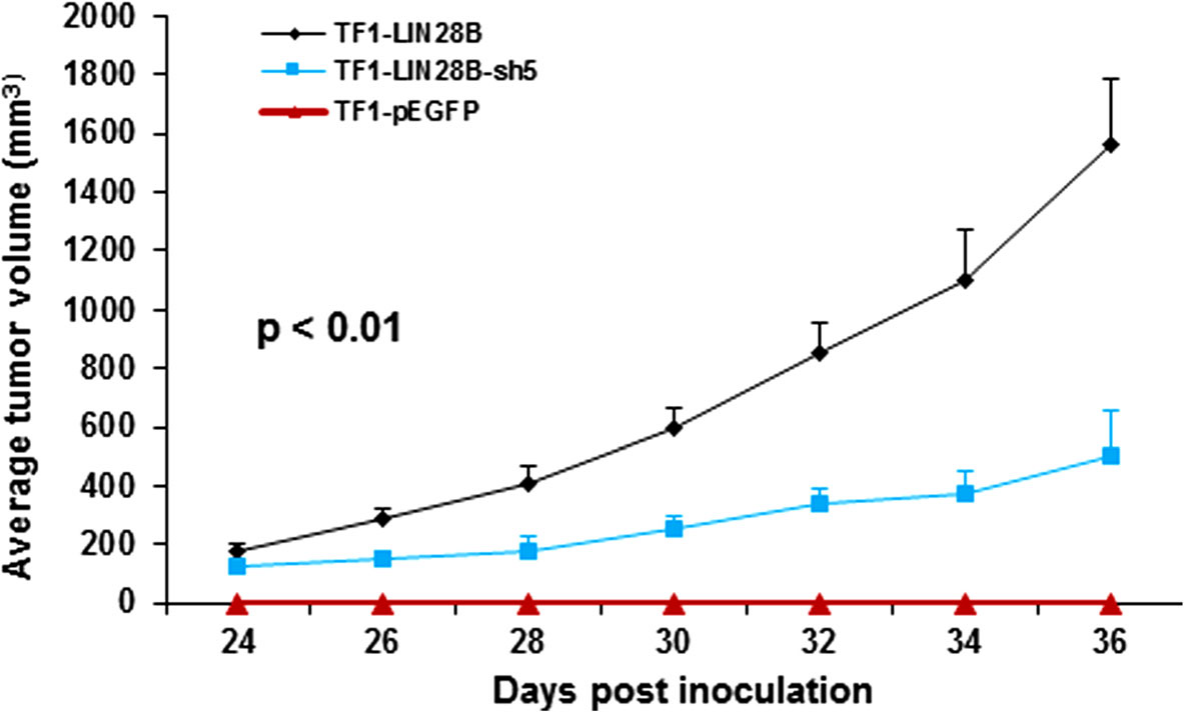
Mouse xenograft models of TF1-pEGFP, TF1-LIN28B, and TF1-LIN28-shRNA5 cells. Three million TF1-pEGFP, TF1-LIN28B, TF1-LIN28B-expressing LIN28B-shRNA5 (TF1-LIN28B-sh5) cells were subcutaneously injected into left inguinal regions of NOD/SCID recipient mice, respectively. The tumor volume was measured by caplier every other day. The tumor growth curves were constructed according to the average tumor volume of each group ± SD (mm^3^) (*n* = 10, *p* < 0.01).

To determine the tumorigenic role of LIN28B in vivo, we used this pair of TF1-pEGFP and TF1-LIN28B cells, as well as TF1-LIN28B cells expressing LIN28B-shRNA5 cells (TF1-LIN28B-sh5) to subcutaneously inject into one side of the inguinal region of NOD/SCID mice. TF1-LIN28B cells formed tumor mass at day 24 post inoculation and progressed rapidly up to 1564.3 ± 220 mm^3^ at day 36 after cell inoculation in immunodeficient mice (Fig. 6). Strikingly, the tumors of TF1-LIN28-sh5 cells developed significantly smaller in size (503.2 ± 150 mm^3^) when compared with TF1-LIN28B tumors (Fig. 6, *p* < 0.01). Notably, TF1-pEGFP cells failed to form any tumor mass in mice (Fig. 6). Therefore, these data suggest that LIN28B confers important oncogenic function in AML cells in vivo.

## Discussion

The RNA-binding protein, LIN28B, has been implicated in various solid tumors and hematological malignancies, including AML [8]. Emerging evidence have suggested LIN28B in contributing to the transformation of cancer stem cells [27, 28]. The most well-studied molecular mechanism of LIN28B in oncogenesis is its ability in regulating let-7 miRNA biogenesis through a TUTase-independent mechanism by sequestering pri-let-7 miRNAs in the nucleoli [29]. The repression of members of let-7 miRNAs could well elicit the de-repression of several oncogenes such as K-Ras, c-Myc, and HMGA2 [30–32], in turn, promoting disease progression.

This study shows that by modulating LIN28B expression could lead to significant changes in cell proliferation and cell cycle. Notably, LIN28B overexpression renders AML cells growth independent of cytokines and enhances tumorigenicity in vivo, suggesting the role of LIN28B in more aggressive tumor phenotype. Reducing LIN28B not only causes AML cells less actively replicating but also induces G_2_/M cell cycle arrest. Interestingly, it has been reported that overexpression of let-7a miRNA could induce a prominent G_2_/M phase arrest in prostate cancer [33].

Numerous human malignancies such as ovarian cancer, lung cancer, hepatocellular carcinoma, and melanoma have been shown to have repression of multiple members of the let-7 family of miRNAs, thus promoting oncogenesis by depressing targets such as c-Myc, Ras, and HMGA2 [10, 34]. In this study, IGF2BP1 has been increased by LIN28B through repression of let-7 miRNA. IGF2BP1 is a novel target gene of let-7 miRNAs. The IGF2BP1 belongs to a conserved family of RNA binding, oncofetal proteins that consist of other isoforms, IGF2BP2, and IGF2BP3 [35]. IGF2BP1 and IGF2BP3 are re-expressed in several aggressive cancers in colorectal and lymphomas with a high incidence of more than 70% [35]. In particularly, IGF2BP1 has been shown to prevent cleavage of MYC mRNA from endonucleases by binding to the CRD (coding region stability determinant) in the MYC open reading frame [36]. Furthermore, overexpression of IGF2BP1 is found to be associated with increased c-MYC and RAS expressions, while loss of IGF2BP1 could induce caspase-3 and PARP-mediated apoptosis in colorectal cancer cell lines [36]. Hence, the regulation of IGF2BP1 plays an important role in LIN28B-mediated oncogenesis. As let-7 miRNA family concurrently regulates several pivotal oncogenic pathways, there is of great interest in re-expression of let-7 miRNAs as a therapeutic option for cancer. Strategies using viral vector, to overexpress let-7 miRNAs or by introducing artificial double-stranded miRNA (mimic of let-7 miRNAs), hold great promising for novel anti-AML therapy.

Studies in stem cell, growth and metabolic disorders, and cancers uncover LIN28/LIN28B as a central regulator of cellular metabolism through let-7-dependent or let-7-independent manner, where LIN28/LIN28B directly binds mRNA of glycolysis and mitochondrial OxPhos enzymes and enhances their translation [37–40]. One of the hallmarks of cancer cells is rapid growth and proliferation by evading suppression signaling [40]. In order to sustain high growth rate, cancer cells often demand more amino acid, nucleotide, ATP, etc., by reprogramming their metabolic pathways [41]. Our gene expression profiling results uncover that LIN28B plays a significant role in wide ranges of metabolic processes of AML cells, involving amino acids, oxoacid, organic acids, carboxylic acids, and neutral amino acids transportation. These organic compounds are mainly utilized in essential metabolic pathways such as glycolysis, tricarboxylic acid (TCA) cycle, and gluconeogenesis [42, 43]. These basic molecules are essential for increased DNA replication, RNA production, and protein synthesis in the LIN28B overexpressing cells. In addition to uncontrolled growth, the other characteristic of AML cells is arrested differentiation. A recent report demonstrates that LIN28/LIN28B regulates stem cell metabolism and facilitate conversion from naive to primed pluripotency [37]. So, it is reasonable to suppose that these altered metabolic pathways induced by LIN28B also contributes to keep AML cells in differentiation blocked states. Particularly, it is worth of highlighting the top most downregulated genes: phosphoserine aminotransferase 1 (PSAT1), phosphoglycerate dehydrogenase (PHGDH), asparagine synthetase (ASNS), serine methyl transferase2 (SHMT2), and cystine-glutamate transporter, SLC7A11 in the LIN28B knockdown cells. These genes have been demonstrated to promote tumorigenesis in a variety type of cancers and associated with poor prognosis [44]. PSAT1, PHGDH, and SHMT2 are three key enzymes in the serine and glycine pathway and enhanced serine and glycine biosynthesis provides sufficient precursors for the synthesis of proteins, nucleic acids, and lipids for highly proliferating cancer cells [45, 46]. Meanwhile, the upregulation of ASNS allows asparagine biosynthesis, making the cancer cells to be less sensitive to asparagine depletion. Hashimoto K et al. demonstrated the importance of decreasing ASNS level for monocytic differentiation in HL-60 cells [47]. On the other hand, Huang. Y et. al showed that the overexpression of SLC7A11 not only mediates cellular uptake of l-alanosine but also confers glutathione-mediated chemoresistance [48]. Thus, these findings open new opportunities for developing better biomarkers for predicting the efficacy of anti-leukemic treatments and selecting optimal drug therapies such as using l-alanosine or other amino acid-related drugs as novel therapeutic strategies like differentiation therapy.

## Conclusions

In summary, our findings demonstrate a pivotal role of LIN28B/let-7/IGF2BP1 in progression of AML and indicate the essential changes in metabolic pathways by LIN28B. Thus, targeting LIN28B or let-7 may be an effective therapeutic option for AML patients.

## Additional files

Additional file 1: Table S1. The sequences of primers for qRT-PCR analysis. (DOCX 15 kb)

Additional file 2: Figure S1. qRT-PCR validation of important metabolism related genes and oncogenes identified through microarray experiments. The values of TF-1a LIN28B-shRNA represent the average of LIN28-shRNA3 and -shRNA5 values. The experiments were triplicated (mean ± SD). (TIF 167 kb)

Additional file 3: Table S2. Gene symbols and their respective mean fold change of LIN28B knocking down TF-1a cells relative to scramble-shRNA treated TF-1a from microarray analysis. (DOCX 19 kb)

Additional file 4: Figure S2. Knockdown of LIN28B induced G_2_/M cell cycle arrest and S phase inhibition in TF-1a cells. Two million of TF-1a-Scramble shRNA, TF-1a-LIN28B-shRNA3, and TF-1a-LIN28-shRNA5 cells were washed in ice-cold PBS, and fixed in 70% cold ethanol for at least 30 minutes. The cell pellets were resuspended in a 1 ml propidium iodide (PI)/RNase staining buffer and incubated for 15 minutes at room temperature, then followed by FACS analysis of cell cycle distributions. Representative images of DNA histogram were shown in (A) and quantification bar figure was presented in (B). This experiment was duplicated. (*n* = 2, mean ± SD, *p < 0.05, **p < 0.01). (TIF 52 kb)

Additional file 5: Figure S3. Cell proliferation assays of TF-1a cells treated with IGF2BP1 specific shRNA3, shRNA4 (left panel) or let-7a mimic, let-7b mimics at indicated time points (*n* = 3, mean ± SD, *p < 0.05). (TIF 128 kb)

## Abbreviations

AML: Acute myeloid leukemia
CR: Complete remission
CSC: Cancer stem cell
HSC: Hematopoietic stem cell
LSC: Leukemia stem cell
