# Supplementary material for: Inhibition of LIN28B impairs leukemia cell growth and metabolism in acute myeloid leukemia
Source: J Hematol Oncol. 2017 Jul 11;10:138. doi: 10.1186/s13045-017-0507-y (PMC5504806; doi:10.1186/s13045-017-0507-y)
Supplement: Supplementary file 1 — The sequences of primers for qRT-PCR analysis. (DOCX 15 kb) [file 13045_2017_507_MOESM1_ESM.docx]

**Table S1. The sequences of primers for qRT-PCR analysis.**
