# Supplementary figures and images for: Inhibition of LIN28B impairs leukemia cell growth and metabolism in acute myeloid leukemia
Source: J Hematol Oncol. 2017 Jul 11;10:138. doi: 10.1186/s13045-017-0507-y (PMC5504806; doi:10.1186/s13045-017-0507-y)

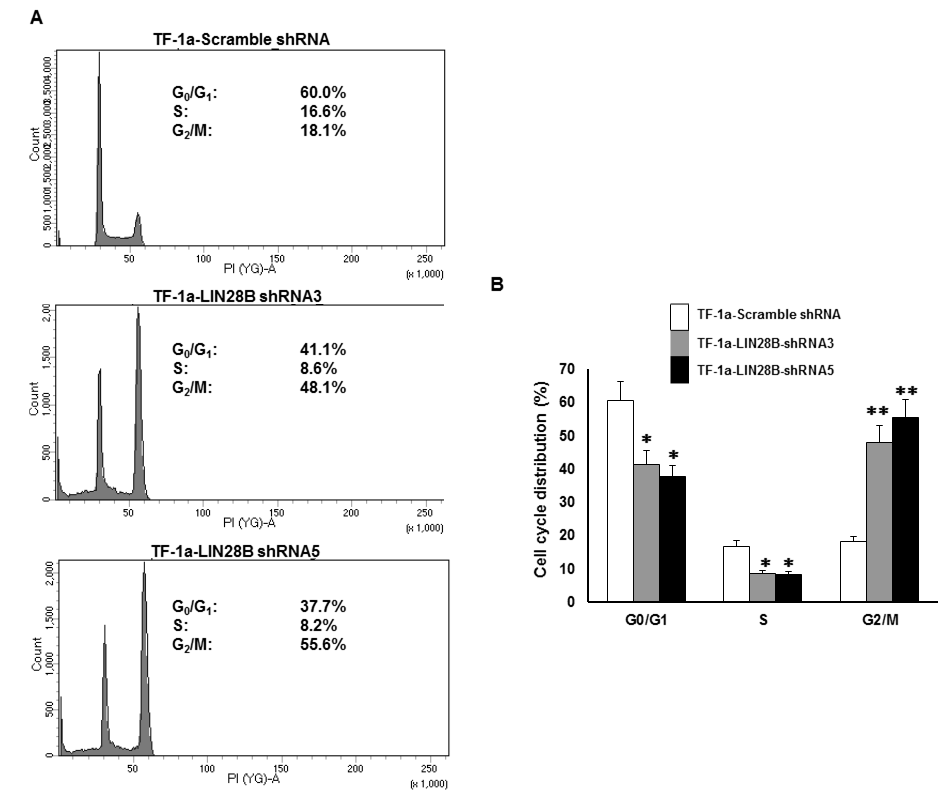

Supplement: Supplementary file 2 — qRT-PCR validation of important metabolism related genes and oncogenes identified through microarray experiments. The values of TF-1a LIN28B-shRNA represent the average of LIN28-shRNA3 and -shRNA5 values. The experiments were triplicated (mean ± SD). (TIF 167 kb) [file 13045_2017_507_MOESM2_ESM.tif]

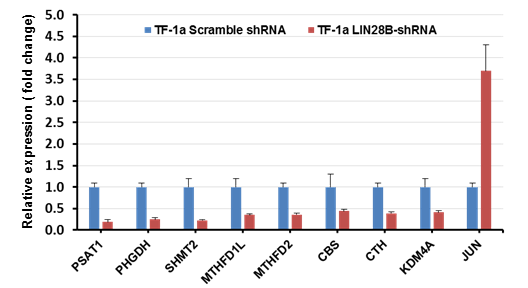

Supplement: Supplementary file 4 — Knockdown of LIN28B induced G2/M cell cycle arrest and S phase inhibition in TF-1a cells. Two million of TF-1a-Scramble shRNA, TF-1a-LIN28B-shRNA3, and TF-1a-LIN28-shRNA5 cells were washed in ice-cold PBS, and fixed in 70% cold ethanol for at least 30 minutes. The cell pellets were resuspended in a 1 ml propidium iodide (PI)/RNase staining buffer and incubated for 15 minutes at room temperature, then followed by FACS analysis of cell cycle distributions. Representative images of DNA histogram were shown in (A) and quantification bar figure was presented in (B). This experiment was duplicated. (n = 2, mean ± SD, *p < 0.05, **p < 0.01). (TIF 52 kb) [file 13045_2017_507_MOESM4_ESM.tif]

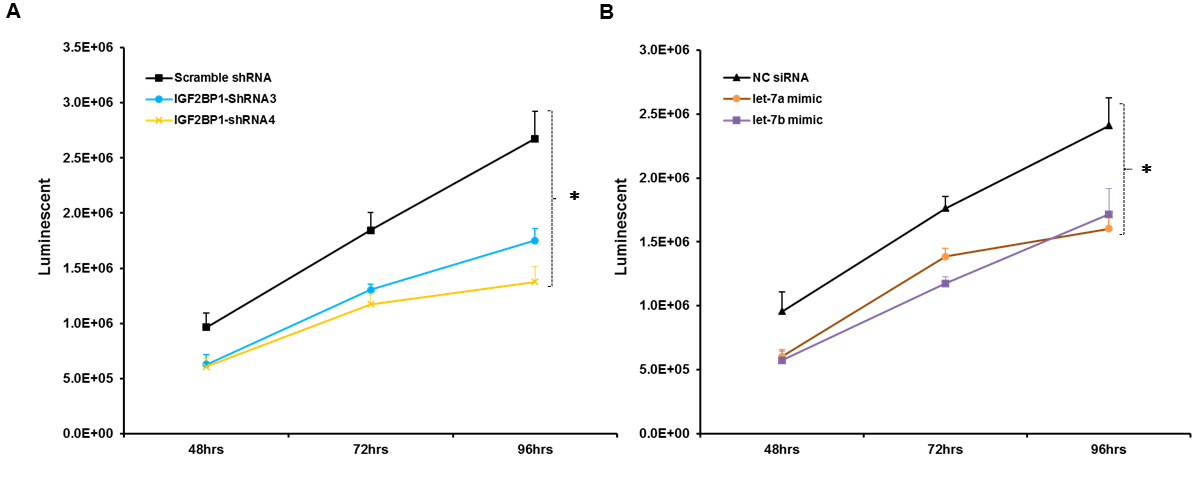

Supplement: Supplementary file 5 — Cell proliferation assays of TF-1a cells treated with IGF2BP1 specific shRNA3, shRNA4 (left panel) or let-7a mimic, let-7b mimics at indicated time points (n = 3, mean ± SD, *p < 0.05). (TIF 128 kb) [file 13045_2017_507_MOESM5_ESM.tif]
