# Supplementary material for: Inhibition of LIN28B impairs leukemia cell growth and metabolism in acute myeloid leukemia
Source: J Hematol Oncol. 2017 Jul 11;10:138. doi: 10.1186/s13045-017-0507-y (PMC5504806; doi:10.1186/s13045-017-0507-y)
Supplement: Supplementary file 3 — Gene symbols and their respective mean fold change of LIN28B knocking down TF-1a cells relative to scramble-shRNA treated TF-1a from microarray analysis. (DOCX 19 kb) [file 13045_2017_507_MOESM3_ESM.docx]

**Table S3: Gene symbols and their respective mean fold change of LIN28B knocking-down TF-1a cells relative to scramble-shRNA treated TF-1a from microarray analysis.**

| Transcripts Cluster Id | Genesymbol | Regulation | Fold change (mean) |
| --- | --- | --- | --- |
| 8136807 | PRSS2\|PRSS3\|PRSS1 | up | 5.798 |
| 8136801 | TRY6\|PRSS2\|PRSS3\|PRSS1 | up | 4.956 |
| 7995783 | MT2A | up | 4.905 |
| 7902721 | CLCA1 | up | 3.687 |
| 7991750 | HBZ | up | 3.167 |
| 8152962 | LRRC6 | up | 2.737 |
| 7995838 | MT1X | up | 2.667 |
| 7951662 | CRYAB | up | 2.654 |
| 8095362 | MT2A | up | 2.499 |
| 8095376 | MT2A | up | 2.474 |
| 8136795 | PRSS1\|PRSS2 | up | 2.406 |
| 7964303 | TAC3 | up | 2.397 |
| 7926239 | OPTN | up | 2.394 |
| 7953291 | CD9 | up | 2.392 |
| 8142524 | TSPAN12 | up | 2.312 |
| 7977615 | RNASE1 | up | 2.278 |
| 8044212 | SULT1C2 | up | 2.256 |
| 8042283 | HSPC159 | up | 2.155 |
| 7995237 | AHSP | up | 2.126 |
| 8001531 | MT1G | up | 2.122 |
| 8100464 | NMU | up | 2.054 |
| 8179519 | HLA-DPB1 | up | 2.050 |
| 8135587 | CAV2 | up | 2.038 |
| 8041644 | PLEKHH2 | up | 1.992 |
| 8046380 | ITGA6 | up | 1.978 |
| 8129231 | FAM184A | up | 1.917 |
| 8083616 | MLF1 | up | 1.908 |
| 8151592 | CA1 | up | 1.869 |
| 7946061 | HBE1 | up | 1.849 |
| 8118594 | HLA-DPB1 | up | 1.833 |
| 8129649 | C6orf192 | up | 1.825 |
| 8179322 | HSPA1A\|HSPA1B | up | 1.803 |
| 8118310 | HSPA1A\|HSPA1B | up | 1.793 |
| 7991766 | HBA1\|HBA2 | up | 1.790 |
| 7991762 | HBA2\|HBA1 | up | 1.789 |
| 8116818 | BMP6 | up | 1.782 |
| 8156905 | TMEFF1 | up | 1.761 |
| 8050160 | MBOAT2 | up | 1.743 |
| 7917037 | CRYZ | up | 1.723 |
| 8174201 | BEX1 | up | 1.718 |
| 7908409 | RGS2 | up | 1.696 |
| 7917347 | DDAH1 | up | 1.692 |
| 8133876 | CD36 | up | 1.672 |
| 8042391 | PLEK | up | 1.653 |
| 7970793 | SLC46A3 | up | 1.646 |
| 7983890 | GCOM1\|GRINL1A | up | 1.643 |
| 7916609 | JUN | up | 1.637 |
| 8103722 | HSP90AA6P | up | 1.621 |
| 7916843 | GNG12 | up | 1.609 |
| 7902512 | DNAJB4 | up | 1.604 |
| 8114030 | KIF3A | up | 1.591 |
| 8133961 | RUNDC3B | up | 1.590 |
| 8147439 | PLEKHF2 | up | 1.586 |
| 8068238 | IFNAR2\|IL10RB | up | 1.581 |
| 7927732 | ARID5B | up | 1.575 |
| 8111915 | SEPP1 | up | 1.553 |
| 8056890 | CHN1 | up | 1.542 |
| 8142307 | PNPLA8 | up | 1.537 |
| 8169519 | WDR44 | up | 1.535 |
| 8022514 | C18orf45 | up | 1.528 |
| 7918157 | VAV3 | up | 1.512 |
| 7928937 | MINPP1 | up | 1.510 |
| 7984517 | GLCE | up | 1.474 |
| 8135378 | PRKAR2B | up | 1.458 |
| 8102800 | SLC7A11 | down | 3.676 |
| 8141150 | ASNS | down | 3.657 |
| 7904433 | PHGDH | down | 3.332 |
| 8156043 | PSAT1 | down | 3.260 |
| 7902290 | CTH | down | 2.827 |
| 8082797 | TF | down | 2.793 |
| 7981290 | WARS | down | 2.589 |
| 7945803 | CARS | down | 2.584 |
| 8121251 | LIN28B | down | 2.345 |
| 7973530 | PCK2 | down | 2.278 |
| 8122724 | ULBP1 | down | 2.158 |
| 7956443 | MARS | down | 2.122 |
| 8154100 | VLDLR | down | 2.076 |
| 8002347 | AARS | down | 1.974 |
| 7956401 | SHMT2 | down | 1.942 |
| 8139656 | GRB10 | down | 1.912 |
| 8003298 | SLC7A5 | down | 1.865 |
| 7914563 | YARS | down | 1.862 |
| 8037835 | SLC1A5 | down | 1.809 |
| 7954436 | LRMP | down | 1.792 |
| 7961829 | BCAT1 | down | 1.785 |
| 7940717 | SLC3A2 | down | 1.771 |
| 8042310 | SLC1A4 | down | 1.765 |
| 7928308 | DDIT4 | down | 1.759 |
| 7962792 | OR10AD1 | down | 1.711 |
| 7900833 | KDM4A | down | 1.705 |
| 8128977 | TUBE1\|C6orf225\|WISP3 | down | 1.703 |
| 8122773 | MTHFD1L | down | 1.697 |
| 8070632 | CBS | down | 1.630 |
| 8132250 | BMPER | down | 1.624 |
| 8038029 | CARD8 | down | 1.616 |
| 8100688 | TMPRSS11F | down | 1.613 |
| 7956426 | INHBE | down | 1.609 |
| 8132070 | GARS | down | 1.577 |
| 7962516 | SLC38A1 | down | 1.573 |
| 8157463 | C9orf91 | down | 1.561 |
| 8153959 | DOCK8 | down | 1.556 |
| 7951565 | ARHGAP20 | down | 1.536 |
| 8031714 | ZNF460 | down | 1.532 |
| 8042830 | MTHFD2 | down | 1.530 |
| 8008151 | IGF2BP1 | down | 1.517 |
| 8057578 | CALCRL | down | 1.483 |
| 8023481 | NARS | down | 1.460 |
